# Captive breeding of *Falco* sp. (Lesser Kestrel, Common Kestrel, Red-footed Falcon) with permanent disabilities in Bulgaria

**DOI:** 10.3897/BDJ.13.e148111

**Published:** 2025-04-08

**Authors:** Rusko Petrov

**Affiliations:** 1 Trakia University, Stara Zagora, Bulgaria Trakia University Stara Zagora Bulgaria; 2 Green Balkans - Stara Zagora NGO, Stara Zagora, Bulgaria Green Balkans - Stara Zagora NGO Stara Zagora Bulgaria

**Keywords:** raptor conservation, birds of prey, ex-situ conservation, captive breeding

## Abstract

Captive breeding of different species of birds offers conservationists a viable option when it comes to boosting wild populations of rare endangered species. Including non-releasable animals with permanent injuries can increase the breeding flock and breeding output. In addition, it provides an opportunity for birds that cannot be released to be included in conservation activities by rearing offspring for release in the wild. Between 2013 and 2024, in Bulgaria in the Wildlife Rehabilitation and Breeding Centre, part of Green Balkans - Stara Zagora NGO, individuals from three species of small falcons were paired for breeding - the pairs included birds with permanent disabilities of Lesser Kestrels, Common Kestrels and Red-footed Falcons. For the study period, 34 Lesser Kestrels - offspring to injured pairs, were reared and released into the wild in the country, together with 172 Common Kestrels and two Red-footed Falcons. The breed-and-release activities contributed to reinforcing the wild populations of the falcon species and to improving the skills and knowledge of aviculturists and veterinarians in Bulgaria dealing with birds of prey.

## Introduction

Previously abundant, in the early 21^st^ centu**r**y, there was no confirmed breeding of Lesser Kestrels (*Falconaumanni*) in Bulgaria (Mateeva et al. 2013). Following the implementation of two projects in which juveniles were released into the wild (both supported by LIFE programme of the EU) (Vasileva et al. 2022b), there currently are three confirmed breeding territories with Lesser Kestrel colonies in the country or 38 pairs (Gradev et al. 2015, Gradev et al. 2021). The Common Kestrel (*Falcotinnunculus*) is the most numerous falcon species in Bulgaria with 4000-7500 breeding pairs (BSPB 2024). The Red-footed Falcon (*Falcovespertinus*) is common as a migrant; however, it has a very small breeding population in the country - estimated at 15-50 breeding pairs for 2006-2009 (Palatitz et al. 2009) or 0-16 in 2020 (Cheshmedzhiev and Stoychev 2024). There are numerous threats to their populations like intensive farming practices (Spasov et al. 2014) amongst others.

Captive breeding programmes for various animal species can provide conservationists with a practical solution to help increase the populations of rare and endangered species in the wild. (IUCN/SSC 2013, McGowan et al. 2016). Incorporating animals with permanent injuries that cannot be released can help boost the captive breeding population and increase breeding success. Additionally, it offers a chance to involve animals with permanent disabilities or handicaps in conservation efforts by raising offspring for release into the wild.

In Bulgaria, in the WRBC, rare endangered species of birds, together with common ones, were breeding and rearing their own and foster offspring as part of the activities of the rescue centre and for various species conservation projects. Captive breeding was instrumental in the processes of recovering the Lesser Kestrel and Saker Falcon populations (Gradev 2021, Lazarova et al. 2021, Petrov et al. 2021, Vasileva et al. 2022a, Vasileva et al. 2022b) and it is underway to play a part in the re-introduction of the Bearded Vulture in the country (Balkans 2024). Breeding individuals with permanent injuries was part of the work of the WRBC; in this way, breeding groups were increased by using birds which have undergone treatment at the clinic, but could not be released (Green Balkans, 2024). This method has already been reported for some of the founding individuals of a Lesser Kestrel captive breeding group in Spain (Alcaide et al. 2009), for example.

The aim of this paper is to present data on the successful breeding in captivity of birds with permanent injuries of the three small falcon species - Lesser Kestrel, Common Kestrel and Red-footed Falcon, implemented in the WRBC in Bulgaria in the period between 2013 and 2024. The information could be beneficial to other rehabilitation and breeding centres and to conservationists dealing with these species.

## Material and methods

### Lesser Kestrels

In the Green Balkans WRBC, for the period 2017 - 2024, there were a total of three breeding pairs of Lesser Kestrels in which one of the partners had a permanent disability - one male had a missing tail and two males were missing a leg. The females were healthy. These pairs were part of the breeding stock of the breed-and-release programme for the restoration of the species in Bulgaria.

### Common Kestrels

From 2013 to 2024, there were between two and eleven pairs of disabled Common Kestrels (both sexes) each season. All the birds in the pairs were patients in the WRBC that could not be released (at all or right away) due to their injuries. They were breeding in captivity and either acted as foster parents or their offspring were released in the wild. Their injuries included wing fractures (16 birds), various wounds (7 birds), broken or missing feathers (6 birds), amputated wing (5 birds), amputated leg (2 birds) and two had fallen in motor oil. Two individuals were physically healthy, but were imprinted on humans after prolonged care in private homes from where they were confiscated. Some were eventually successfully released after a rehabilitation period - and after successful feather growth, osteosynthesis or healing of wounds.

### Red-footed Falcons

There was one pair of Red-footed Falcons in 2020 and 2021, consisting of a female with a missing wing and a male which had a fracture of the radius and ulna bones. For the period 2022-2024, there was also only one pair made up of the same female falcon and a male which was missing the last digits of one wing. The three birds were part of the admitted patients in the WRBC which, as the Common Kestrels could not be released in the wild due to their injuries, they were left in captivity.

### Captive breeding

The Wildlife Rehabilitation and Breeding Centre (WRBC) is part of Green Balkans - Stara Zagora NGO, is located in the town of Stara Zagora, Bulgaria and it is the only facility of its kind in the country. It deals with rescue and rehabilitation of injured wildlife - birds and small mammals and reptiles and has breed-and-release programmes for rare and endangered bird species. There are various aviaries tailored to different species requirements, in addition to a combined structure for the *ex situ* breeding, adaptation and release of Lesser Kestrels. The Lesser Kestrel cage was hexagonal, with a diameter of 3.5 m and a height of 3.5 m. The Common Kestrel and Red-Footed Falcon aviaries measured 2 m by 3.5 m and had a height of 2 m.

The Lesser Kestrels chose their partners themselves as they were all in one cage and have the freedom to choose partners and nestboxes. The Common Kestrels and Red-Footed Falcons we paired as soon as a non-releasible individual was accepted in the WRBC and the veterinary physicians had determined their injuries did not affect breeding ability. The pairs were left to incubate naturally, with the exception of some of the Lesser Kestrels for which the first clutch, if laid early in the season, was taken to an incubator, stimulating them to lay a second one - high productivity was aimed at throughout the re-introduction programme (Gradev 2021). Some of the Common Kestrel pairs had also laid a second clutch unassisted. The birds were monitored directly via a one-way mirror in the (Lesser Kestrel) aviary and CCTV cameras (present in all birds’ aviaries and nesting boxes) and their behaviour, courtship and breeding were recorded, noting each bird’s ring number for identification.

### Data processing

We collected data on each pairs’ number of eggs, fertile eggs, hatched chicks and released offspring. Parameters described by Cheylan (1981) - clutch size (number of eggs related to the number of pairs), brood size (number of hatchlings related to the number of pairs) and breeding success (number of released chicks related to the number of pairs) were evaluated. The data were processed using SPSS Statistics (SPSS-Inc., 2019, Chicago, USA). The information was presented in spreadsheets using Microsoft Excel.

## Results

The captive breeding of injured pairs resulted in 97% of hatched Lesser Kestrels being successfully released in the wild, 98% of the Common Kestrels and 75% of the Red-footed Falcons, respectively. The parameters clutch size, brood size and breeding success for the three falcon species were presented in Table 1.

## Discussion

The role of zoos and specialised centres in *ex situ* activities like captive breeding has proven instrumental for the conservation of some endangered species of birds (Khan and Murn 2011). Successful cases include the Peregrine Fund’s notorious recovery of the California Condor (*Gymnogypscalifornianus*) and Peregrine and Aplomado Falcons (*Falcoperegrinus*, *Falcofemoralis*) in the USA (Fund 2024); recovery of the Mauritius Kestrel (*Falcopunctatus*) (Jones et al. 1995); re-introduction of captive-bred Bearded Vultures (*Gypaetusbarbatus*) in the Alps (Waldvogel et al. 2017), amongst many more.

Compared to Gradev (2021)'s mean values of the Lesser Kestrels in the wild colony in Levka, Bulgaria, in the WRBC, we had larger clutch sizes (4.58 compared to 3.818) and slightly higher breeding success (2.47 compared to 2.01). The brood size in our study was smaller (2.53 vs. 3.818). Compared to Rodríguez et al. (2013) - clutch size of 4.25 and breeding success of 1.54 (or 2.5-3 fledglings per successful pair), our values were comparable or higher than those of the urban colony in Spain. This could be the result of the artificial incubation performed for this species in the WRBC - taking a pair’s first clutch to place it in an incubator stimulates the laying on a second one and, thus, increasing the overall clutch size. The mean clutch size of the Common Kestrels in our study was 4.30, a bit less than that of our Lesser Kestrels and less than the mean of Common Kestrels in the wild in Hungary (in artificial nests) - 5.05 (Kotymán et al. 2015). Second clutches of Common Kestrels have been observed in the wild in the Czechia by Poprach and Dusík (2024) and they also noted they occur in captivity with no assistance, which was true for our Common Kestrel pairs. Second breeding events are explained by the abundance of food, which can also be the case in breeding centres. However, the mean number of fledglings in our study - 2.33, was considerably lower compared to the one of wild kestrels in nest-boxes in Poland - 4.2 (Rzępała et al. 2022). It should be taken into account they originated from the wild and they only started breeding after undergoing treatment in the WRBC and there could potentially be unknown factors accounting for their lower fertility. The mean clutch size of the Red-footed Falcons in our study - 2.00, was also significantly lower than that reported in the wild in Hungary (in artificial nests) - 3.38 (Kotymán et al. 2015) and that of the other falcon species in the WRBC. A possible explanation could be the fact that they are a facultative colony breeder in the wild (Palatitz et al. 2015), whereas in the WRBC, there was only one breeding pair per season.

## Conclusions

Captive breeding birds with permanent injuries of three small falcon species - Lesser Kestrel, Common Kestrel and Red-footed Falcon, led to increased numbers of young birds released into the wild in Bulgaria. The method could be used in breeding strategies in order to increase the breeding group and released offspring, aiding breed-and-release conservation programmes by including non-releasable birds. In addition, it can aid capacity building of aviculturists and veterinarians dealing with birds of prey. Potential individual conditions of the birds related to lower fertility or inability to reproduce should be considered.

## Figures and Tables

**Table 1.** Clutch size, brood size and breeding success for the disabled pairs of the three falcon species in the WRBC for the study period.

		Pairs	Eggs	Fertilised	Hatched	Released	Clutch size	Brood size	Breeding success
Lesser Kestrels	Mean ± SD	2 ± 0.89	9 ± 4.05	6.67 ± 4.72	5.83 ± 4.17	5.67 ± 3.93	4.58 ± 0.92	2.53 ± 1.37	2.47 ±-1.32
	N	6.00	6.00	6.00	6.00	6.00	6.00	6.00	6.00
	Min-max	1-3	4-15	0-12	0-11	0-10	3-5.50	0-3.67	0-3.50
	Range	2.00	11.00	12.00	11.00	10.00	2.50	3.67	3.50
Red-Footed Falcons	Mean ± SD	1 ± 0.00	2±1.41	1.75 ± 1.26	1 ± 1.15	0.75 ± 0.96	2 ± 1.41	1 ± 1.15	0.75 ± 0.96
	N	4.00	4.00	4.00	4.00	4.00	4.00	4.00	4.00
	Min-max	1-1	0-3	0-3	0-2	0-2	0-3	0-2	0-2
	Range	0.00	3.00	3.00	2.00	2.00	3.00	2.00	2.00
Common Kestrels	Mean ± SD	6.17 ± 2.62	27.17 ± 15.99	15.58 ± 9.95	14.58 ± 8.72	14.33 ± 8.88	4.30 ± 1.39	2.38 ± 0.97	2.33 ± 0.99
	N	12.00	12.00	12.00	12.00	12.00	12.00	12.00	12.00
	Min-max	2-11	7-54	4-36	4-32	4-32	1.4-6.2	0.8-4	0.8-4
	Range	9.00	47.00	32.00	28.00	28.00	4.80	3.20	3.20
Total	Mean ± SD	4.09 ± 3.05	17.64 ± 16.06	10.64 ± 9.53	9.73 ± 8.75	9.50 ± 8.79	3.96 ± 1.55	2.17 ± 1.20	2.08 ± 1.21
	N	22.00	22.00	22.00	22.00	22.00	22.00	22.00	22.00
	Min-max	1-11	0-54	0-36	0-32	0-32	0-6.20	0-4	0-4
	Range	10.00	54.00	36.00	32.00	32.00	6.20	4.00	4.00
	p	0.00	0.00	0.01	0.01	0.01	0.01	0.09	0.04
